# Carbapenemase-producing *Klebsiella pneumoniae* and Hematologic Malignancies

**DOI:** 10.3201/eid2007.130094

**Published:** 2014-07

**Authors:** Livio Pagano, Morena Caira, Enrico Maria Trecarichi, Teresa Spanu, Roberta Di Blasi, Simona Sica, Maurizio Sanguinetti, Mario Tumbarello

**Affiliations:** Author affiliation: Catholic University of the Sacred Heart, Rome, Italy

**Keywords:** Key words: *Klebsiella pneumoniae*, carbapenemases, hematologic malignancies, Italy

**To the Editor:** Until a few years ago, the most frequent microbiologically documented cause of severe bloodstream infections among patients with hematologic malignancies was gram-positive bacteria (*1*). However, over the years, gram-negative bacteria have become the main infectious cause of death among patients with hematologic malignancies, and rates of different phenotypes associated with antimicrobial drug resistance are increasing (*2*). This trend could be the result of increasing empirical use of antimicrobial drug therapy and prophylaxis and use of new, more effective antimicrobial drugs. In particular, over the past few years at our hospital (Agostino Gemelli Teaching Hospital, Rome, Italy), we have observed a progressive increase in bloodstream infections caused by *Klebsiella pneumoniae* carbapenemase–producing *K. pneumoniae* (KPC-Kp), which are responsible for a dramatic new scenario.

We reviewed records of all patients affected by hematologic malignancies who were admitted to the hospital hematology department from January 2009 through December 2012 and in whom a bloodstream infection caused by gram-negative bacteria developed. A KPC-Kp bloodstream infection was defined as a bloodstream infection documented on the basis of blood culture positivity (at least 1 specimen) for a KPC-Kp strain and clinical signs of systemic inflammatory response syndrome. The Vitek 2 system (bioMérieux. Firenze, Italy) was used for isolate identification and antimicrobial drug susceptibility testing; PCR and sequencing, as previously described, was used to identify *bla_KPC_* genes (*3*). Antibiograms were reported 72–120 hours (median 76 hours) after onset of bloodstream infection. Death was considered attributable to infection for patients who died within 2 weeks after the first positive blood culture and for whom other potential causes of death could be excluded.

During the study period, we detected 147 bloodstream infections caused by gram-negative bacteria, 38 (25%) of which were caused by *K. pneumoniae*; of these, 26 (18%) were caused by KPC-Kp. We did not identify any episodes of recurrent KPC-Kp bloodstream infection. We did document a progressive, exponential increase in infections caused by KPC-Kp. No KPC-Kp cases were documented until 2009, and cases increased from only 1 case in 2010 to 12 cases in 2012 (Table). The incidence of KPC-Kp among all gram-negative causes of bloodstream infections increased significantly from 2009–2010 (1/69, 1.4%) to 2011–2012 (25/78, 32.1%) (p<0.0001). Most patients with KPC-Kp bloodstream infection had acute myeloid leukemia (14, 53.8%); others had non-Hodgkin lymphoma (4, 15.4%), acute lymphoid leukemia (3, 11.6%), Hodgkin lymphoma (2, 7.8%), myeloproliferative disease (1, 3.8%), myelodysplastic syndrome (1, 3.8%), or aplastic anemia (1, 3.8%). At time of bloodstream infection onset, 19 (73.1%) of 26 patients were markedly neutropenic (<500 × 10^9^ neutrophils/mL for >10 days); almost half (12/26, 46.1%) of these patients experienced complete remission during the course of consolidation therapy or were receiving initial chemotherapy. Among KPC-Kp isolates, 80.8% were susceptible to colistin, 69.2% to tigecycline, and 65.4% to gentamicin. The overall KPC-Kp bloodstream infection–attributable mortality rate was 57.6% (15/26), which was significantly higher than that for bloodstream infections caused by gram-negative bacteria other than KPC Kp (17/121, 14%; p<0.0002) and for bloodstream infections caused by non–KPC-Kp (2/12, 16.7%; p = 0.02) (Table).

**Table:** Prevalence and attributable mortality rate of BSI, Rome, Italy, 2009–2012*

Year

Any gram-negative bacteria

Non-KPC–producing *K. pneumoniae*

KPC-producing *K. pneumoniae*

BSI, no.

Deaths, no. (%)

BSI, no.

Deaths, no. (%)

BSI, no.

Deaths, no. (%)

2009

30

5 (16.6)

1

0

0

0

2010

39

7 (17.9)

2

0

1

1 (100)

2011

41

9 (24.3)

5

1 (20)

13

7 (53.8)

2012

37

11 (29.7)

4

1 (25)

12

7 (58.3)

Total

147

32 (21.7)

12

2 (16.6)

26

15 (57.6)

*BSI, bloodstream infections; KPC, *Klebsiella pneumonia*e carbapenemase.

Despite tailoring of antimicrobial drug therapy to antibiogram results, the KPC-Kp bloodstream infection–attributable mortality rate was high. For ≈50% of patients, therapy consisted of combinations of >2 antimicrobial drugs with in vitro activity against the KPC-Kp isolate. Outcomes are reportedly better after this therapy than after monotherapy (*3*,*4*). 

In our opinion, the high mortality rate related to KPC-Kp bloodstream infections in patients with hematologic malignancies could be related to various factors. First, patients with hematologic malignancies usually receive antimicrobial drugs recommended for the management of fever in immunocompromised patients with cancer but rarely receive empirically administered drugs active against KPC-Kp bloodstream infections. The delay in appropriate antimicrobial treatment reportedly has a strong negative effect on patient outcomes (*5*). Second, KPC-Kp isolates may not be susceptible to the antimicrobial drugs generally considered as the therapy of choice for such infections. In our study, the rates of nonsusceptibility to colistin, tigecycline, and gentamicin were 19%, 31%, and 35%, respectively. Third, many patients have severe clinical conditions caused by hematologic malignancy and other concurrent conditions (e.g., renal failure, heart disease).

In conclusion, in areas where KPC-Kp is endemic, progress in treating hematologic malignancies could be slowed by the emergence of severe KPC-Kp infections. In these settings, the early identification of patients likely to be colonized and/or infected by KPC-Kp strains represents a major step toward preventing and containing the spread of these strains among hospitalized patients. Policies on empirical treatment might need to be revised, depending on the possibility of serious infections caused by carbapenem-resistant *Enterobacteriaceae.*
